# Enhanced Gas Sensitivity of Au-Decorated Flowery WSe_2_ Nanostructures

**DOI:** 10.3390/nano12234221

**Published:** 2022-11-27

**Authors:** Xia Zhang, Qiuhong Tan, Qianjin Wang, Peizhi Yang, Yingkai Liu

**Affiliations:** 1 College of Physics and Electronic Information, Yunnan Normal University, Kunming 650500, China; 2 Yunnan Provincial Key Laboratory for Photoelectric Information Technology, Yunnan Normal University, Kunming 650500, China; 3 Key Laboratory of Advanced Technique & Preparation for Renewable Energy Materials, Ministry of Education, Yunnan Normal University, Kunming 650500, China

**Keywords:** flowery WSe_2_, Au nanoparticle, alcohol detection, gas sensors

## Abstract

With the continuous improvement in material life, people are paying more and more attention to air quality; therefore, it is critical to design efficient and stable gas sensor devices. In this work, a flowery WSe_2_ nanostructure and its nanocomposite (Au@WSe_2_) decorated with Au nanoparticles were fabricated by the hydrothermal method. The performance of a resistive sensor with flowery WSe_2_ and Au@WSe_2_ sensors was evaluated by detecting volatile organic compounds such as ethanol, isoamylol, n-butyl alcohol, isopropanol, isobutanol and n-propanol. The results show that Au-nanoparticle-decorated flowery WSe_2_ can decrease the optimal working temperature from 215 °C to 205 °C and significantly enhance the response of flowery WSe_2_. The response values to isoamylol are the highest (as high as 44.5) at a low gas concentration (100 ppm), while the response values to ethanol are the highest (as high as 178.5) at a high gas concentration (1000 ppm) among the six different alcohols. Moreover, the response is steady and repeatable. The results demonstrate that the Au@WSe_2_ substrate has good responsiveness and selectivity, which makes it a promising candidate for gas detection.

## 1. Introduction

Gas sensors are playing an increasingly vital role in life [1], industry environmental protection [2], combustion control [3], automobile exhaust detection [4], drunk driving inspection, anesthesia monitoring [5,6] and other fields. Within the transition metal disulfide (TMD) family [7], the novel graphene-like layered [8,9] material WSe_2_ [10] has recently received much attention as an emerging nanomaterial due to its outstanding properties, such as a large volume ratio, excellent electrical conductivity, sensitive surface, small bandgap, and high stability [11,12]. The WSe_2_ material proved to be a relatively stable semiconductor among TMD materials, with an indirect bandgap of about 1.2 eV [13], which can be used as an effective material for field-effect transistor channels [12,14].

In recent years, lots of gas sensors based on noble metal and semiconductor composite nanomaterials have been investigated since the noble metal nanoparticles (NPs) can improve the adsorption capacity of the gas-sensitive material surface to the target gas molecules and thus change the resistance. For example, Liu et al. [15] reported that the sensitivity of Au@SnO_2_ was three times higher than that of the pure SnO_2_ sensor and exhibited an excellent response/recovery time. Peng et al. [16] decorated a ZnO nanostructure with 6% Au NPs and found that the composite sensor has about a nine-fold enhancement in its gas response to 100 ppm acetone at 280 °C compared to pristine ZnO. Li et al. [17] reported that the sensitivity of the Au@LaFeO_3_ gas sensor to 100 ppm ethanol is 27 times higher than that of the pure LaFeO_3_ sensor at an optimal operating temperature. In our previous work, we also found that the response of the Au@CuO sensor is seven times higher than that of the pure CuO sensor exposed to 1000 ppm ethanol [18]. Therefore, noble metal NPs can improve the sensitivity of the gas sensor as well as its response/recovery characteristics. To the best of our knowledge, a gas sensor based on the Au-NP-decorated flowery WSe_2_ nanostructure has not been reported yet.

In this paper, to investigate the gas sensitivity of flowery WSe_2_ nanostructures with/without Au NP decoration, six different alcohols were tested before and after the Au decoration of the flowery WSe_2_ sensor. The results indicated that the response to the six alcohols was enhanced after the Au NP decoration of the flowery WSe_2_ sensor, in which the response of Au@WSe_2_ nanocomposites (NCs) to ethanol, n-butyl alcohol and n-propanol were significantly enhanced compared to the pure flowery WSe_2_ sensor. At a low concentration of gas (100 ppm), the response of Au@WSe_2_ NCs to isoamylol displayed the largest response value (~45). However, at a high concentration of gas (1000 ppm), the response of Au@WSe_2_ NCs to ethanol displayed the largest response value (~178.5). The results show that Au@WSe_2_ NCs have a better response and selection performance as well as higher sensitivity to the target gas at the optimal temperature.

## 2. Experimental Section

Hydrothermal synthesis is considered an effective preparation method to fabricate semiconductor nanostructures due to its advantages of low cost, easy operation and good dispersibility [19]. In this work, we synthesized flowery WSe_2_ and Au@WSe_2_ NCs by hydrothermal synthesis and layer-by-layer self-assembly technology for alcohol gas detection.

### 2.1. Chemicals and Materials

Se (purity ≥ 99.9%), Na_2_WO_4_ (purity ≥ 99.0%), NaBH_4_ (purity ≥ 98.0%), NaBH4 (purity ≥ 98%), HAuCl_4_·4H_2_O, Ethanol (purity ≥ 99.5%), Isoamylol (purity ≥ 99.7%), N-butyl Alcohol (purity ≥ 99.5%), Isopropanol (purity ≥ 99.5%), Isobutanol (purity ≥ 99.5%) and n-propanol (purity ≥ 99.5%) were all purchased from Tianjin Sailboat Chemical Reagent Technology Co., Ltd. (Tianjin, China).

### 2.2. The Synthesis of Flowery WSe_2_ and Au@WSe_2_ NCs

**Figure 1:**
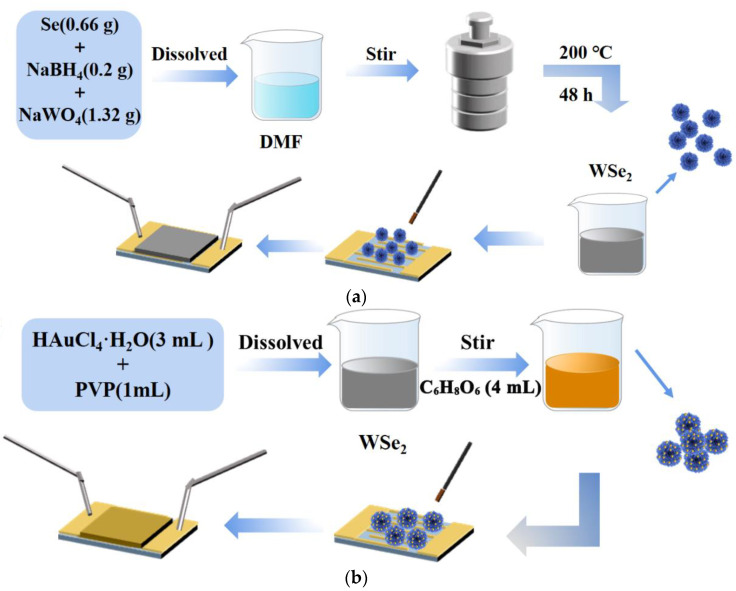
Diagram of prepared (**a**) WSe_2_ and (**b**) Au@WSe_2_ NC sensors.

Flowery WSe_2_ was prepared by a one-step solvothermal method using Se and NaWO_4_ as raw materials, NaBH_4_ as a reducing agent and *N, N*-dimethylformamide (DMF) as a solvent. Se (0.66 g, 0.0084 mole) and NaBH_4_ (0.2 g, 0.0053 mole) were dispersed sequentially into DMF, stirred uniformly to obtain a mixed solution, which was transferred to a 100 mL reaction kettle and reacted at 200 °C for 48 h. The reaction product (black material) was collected by filtration and then washed several times with deionized water and ethanol, followed by drying to obtain the flowery WSe_2_ nanostructures. The specific process is shown in Figure 1a. During the synthesis of Au@WSe_2_ NCs, an appropriate amount of WSe_2_ powder was dispersed in deionized water, and then 3 mL of PVP aqueous solution and 1 mL of HAuCl_4_·H_2_O solution were added successively and fully stirred for 3 min, and then 4 mL of C_6_H_8_O_6_ with a concentration of 0.01mol/L was quickly added with a syringe as a reducing agent solution. After stirring for 3 h, the mixed solution was centrifuged and dried to obtain Au@WSe_2_ NCs, as shown in Figure 1b.

### 2.3. Construction of the Sensors

The preparation details of the gas sensors are as follows. Firstly, the Au electrode surfaces were cleaned with acetone, ethanol and deionized water. Then, 0.002 g of sample powder and 100 μL of deionized water were mixed into a grinding bowl and ground for 2 min, and 8–10 μL of the extracted mixed sample solution was uniformly smeared onto 15 mm × 10 mm Au electrodes with a brush [20]. The electrode line width and electrode spacing were about 0.5 mm and 1 mm, respectively (as shown in Figure S1a). The areas of sensitive layers of WSe_2_ and Au@WSe_2_ gas sensors were both 10 mm × 10 mm (as shown in Figure S1b). Finally, the gold electrode coated with the sample was placed on a hot table and heated at 230 °C for 24 h to test the gas sensor.

### 2.4. Measurement

The sensing properties of the gas sensors were tested using a gas-sensing analysis system (Beijing Elite Tech Co., Ltd., Beijing, China) at the desired gaseous volatile organic compound (VOC) concentrations. The whole process was completed in the CGS-1TP system, which is composed of the main engine, heating platform and gas chamber. The sensor was placed on the heating platform in the closed chamber, and the loop between the electrode and the system was connected. The evaporation temperature of the pan was set to the same value as that of the VOC. When the temperature of the heating platform reached the set value, the sensing resistance dropped to a constant value. The volatile organic solution was then injected into the chamber’s evaporating dish, and the resistance of the device was converted into a visible pattern on the system’s computer. The experimental data were collected by an intelligent gas-sensitive analysis system.

## 3. Results and Discussion

### 3.1. Characterizations

**Figure 2:**
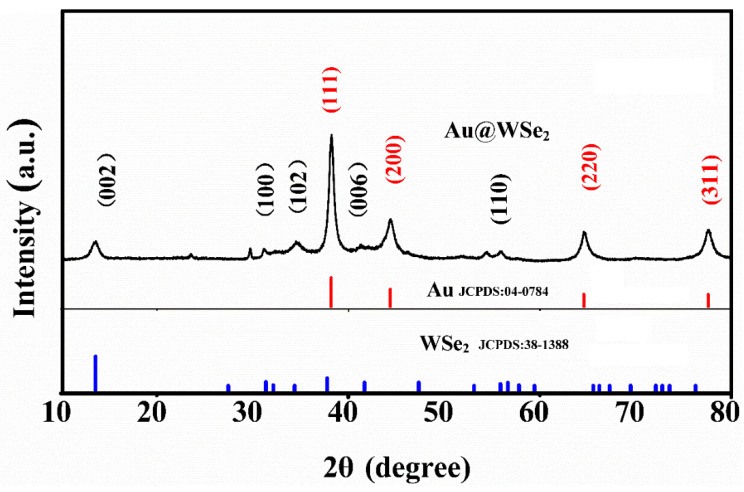
XRD patterns of Au@WSe_2_ NCs.

Figure 2 shows the X-ray diffraction (XRD) patterns of Au@WSe_2_ NCs. It can be seen that some characteristic diffraction peaks correspond to the crystal planes (002), (100), (102), (103), (006) and (110) according to the standard PDF card (JCPDS 38-1388) of the hexagonal WSe_2_ crystal, and some characteristic peaks correspond to the crystal planes (111), (200), (220) and (311) according to the standard PDF card (JCPDS 04-0784) of Au. No additional characteristic diffraction peaks are observed, indicating that flowery WSe_2_ was successfully decorated with Au NPs, forming a composite [21]. The sharp and clear diffraction peaks indicate that the prepared Au@WSe_2_ NCs have good crystallinity.

**Figure 3:**
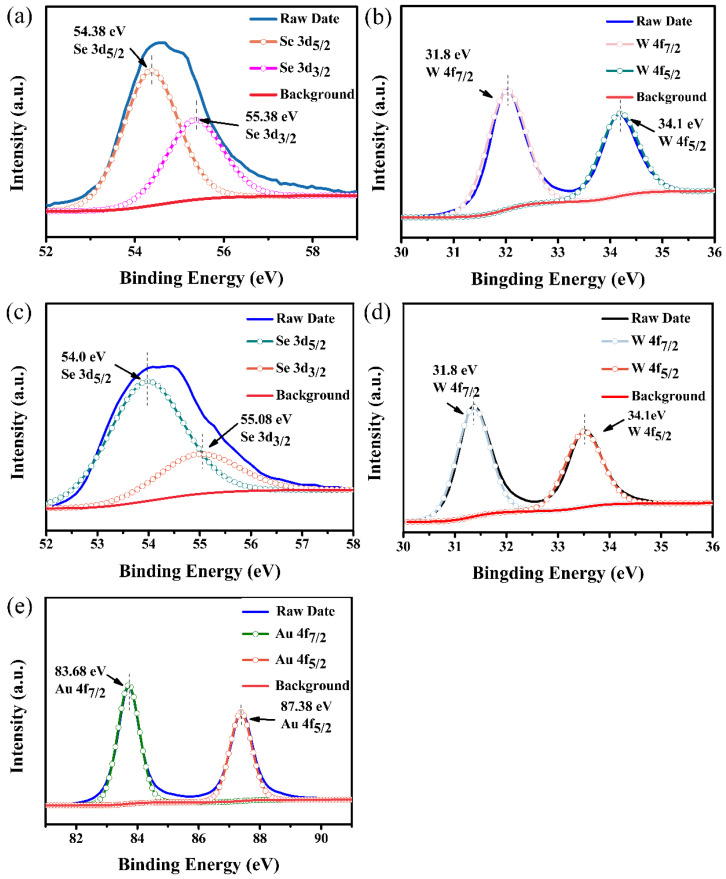
(**a**,**b**) XPS analysis of flowery WSe_2_; (**c**–**e**) XPS analysis of Au@WSe_2_ NCs.

Figure 3 shows the X-ray photoelectron spectroscopy (XPS) spectra of flowery WSe_2_ and Au@WSe_2_ NCs. In order to study each peak of the spectrum more accurately, we fitted the peaks of the main elements with a Gaussian distribution. For pure flowery WSe_2_, the Se 3d spectrum is divided into 54.38 eV and 55.38 eV [22] (as shown in Figure 3a), which correspond to the 3d_5/2_ and 3d_3/2_ orbitals of Se [23], respectively. The spectral peaks for the W 4f_7/2_ and W 4f_5/2_ doublets are located at 31.8 eV and 34.1 eV [24] (as shown in Figure 3b), respectively, which are consistent with the previously reported values for pure WSe_2_ [25]. Figure 3c–e show the XPS energy spectra of Se, W and Au in Au@WSe_2_ NCs. It can be seen in Figure 3c that the binding energies of the two different peaks located at 54.0 eV and 55.08 eV are Se 3d_5/2_ and Se 3d_3/2_, respectively. Compared with the peak position of Se 3d of pure flowery WSe_2_, the peaks of Se 3d_5/2_ and Se 3d_3/2_ in the Au@WSe_2_ NCs are shifted by 0.38 eV and 0.3 eV, respectively. The binding energies of the two initial peaks of W 4f_7/2_ and W 4f_5/2_ are still located at 31.8 eV and 34.1 eV, respectively, as shown in Figure 3d. Figure 3e shows the XPS image of Au 4f. The binding energies for the Au 4f_7/2_ and Au 4f_5/2_ doublets are located at 83.68 eV and 87.38 eV, respectively, which is in good agreement with previous reports [18], indicating that flowery WSe_2_ was successfully decorated with Au NPs without introducing impurities.

**Figure 4:**
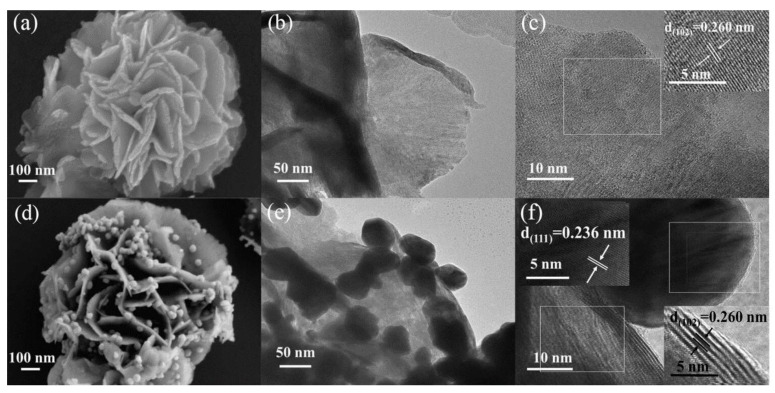
(**a**–**c**) SEM and HRTEM images of flowery WSe_2_; (**d**–**f**) SEM and HRTEM images of Au@WSe_2_ NCs.

In order to further study the microstructure and interfacial state of the fabricated WSe_2_ samples, scanning electron microscopy (SEM), transmission electron microscopy (TEM) and high-resolution TEM (HRTEM) were performed. Figure S2 shows an overview image of an ensemble of the flowery WSe_2_ nanostructures. Figure 4a illustrates that the synthesized WSe_2_ has a loose three-dimensional flower-like structure, which is formed by two-dimensional layered nanosheets. As can be seen in Figure 4b, flowery WSe_2_ only curled at the petal edge, which increased the thickness of the layer. This structure not only provides a larger specific surface area but also provides more active sites for the gas to react on the material surface. Moreover, the coiled petal structure gives the stacked three-dimensional flowery structure a larger interstitial surface and exposes more active sites at the edges, which provides the possibility of rapid gas adsorption and desorption [26]. Figure 4c shows obvious lattice fringes on the flowery WSe_2_ surface with a lattice spacing of 0.260 nm, which matches the lattice plane of (102). Flowery WSe_2_ was reduced to Au@WSe_2_ NCs by the in situ reduction of the HAuCl_4_·4H_2_O solution, as displayed in Figure 4d. As can be seen, Au NPs are tightly attached to the surface of flowery WSe_2_. Further analysis by TEM indicated that the diameter of the Au NPs is about 40 nm, as shown in Figure 4e. The HRTEM images show that the lattice fringes of Au (111) and WSe_2_ (102) planes were observed with lattice spacings of 0.236 and 0.260 nm (as shown in Figure 4f), respectively, indicating that the Au NPs and flowery WSe_2_ are well combined in the Au@WSe_2_ NCs.

### 3.2. Gas-Sensing Properties

**Figure 5:**
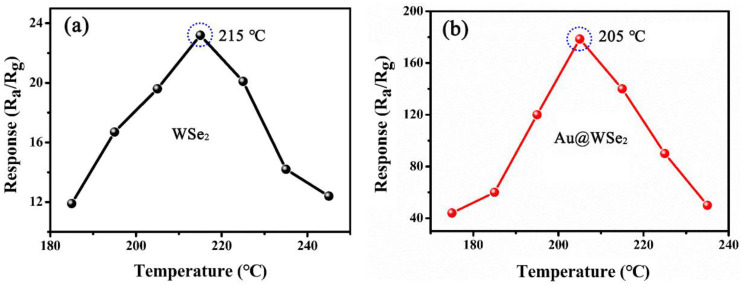
The response vs. temperature of (**a**) pure flowery WSe_2_ and (**b**) Au@WSe_2_ NCs to ethanol at 1000 ppm, respectively.

Figure 5 shows the response curve of pure flowery WSe_2_ and Au@WSe_2_ NC sensors to ethanol at a concentration of 1000 ppm as a function of temperature (T) [27]. The response of the sensor can be calculated by S = R_a_/R_g_, where R_g_ is the resistance of the target gas, and R_g_ is the resistance of the sensor in the target gas [28]. Obviously, the optimal working temperature was reduced from 215 °C to 205 °C, and the response was effectively improved when flowery WSe_2_ was decorated with Au NPs, which is beneficial in prolonging the service life as well as reducing the energy consumption of the device.

**Figure 6:**
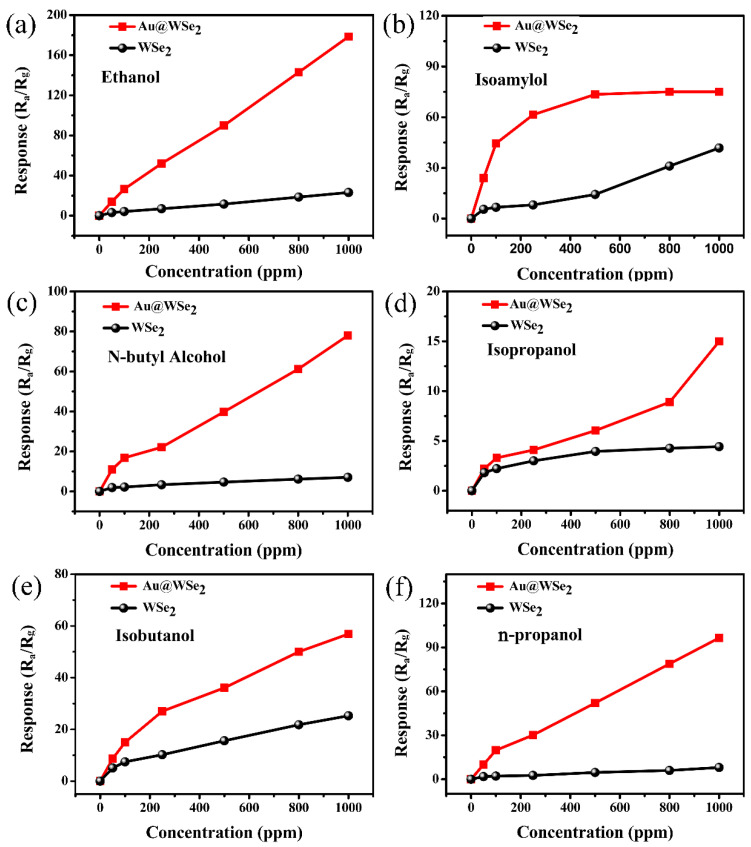
The responses of pure flowery WSe_2_ and Au@WSe_2_ NC sensors to diverse gases at various concentrations. (**a**) Ethanol, (**b**) isoamylol, (**c**) n-butyl alcohol, (**d**) isopropanol, (**e**) isobutanol and (**f**) n-propanol.

**Figure 7:**
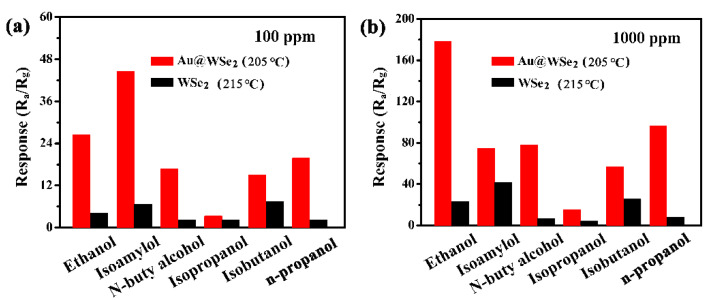
Comparison of sensing performance of Au@WSe_2_ NCs sensor for six alcohols at (**a**) 100 ppm and (**b**) 1000 ppm.

The sensing properties for six different alcohols (ethanol, isoamylol, n-butyl alcohol, isopropanol, isobutanol and n-propanol) were investigated when using the flowery WSe_2_ sensor with/without Au NP decoration. Figure 6 shows the response of pure flowery WSe_2_ and Au@WSe_2_ NC sensors to different gas concentrations at the optimal operating temperature. The results show that the response to the six different alcohols increases with the increase in gas concentration. The response of flowery WSe_2_ was significantly enhanced when it was decorated with Au NPs. At a low gas concentration (100 ppm), the enhancement effect toward ethanol, isoamylol, n-butyl alcohol and n-propanol was particularly significant: their response values were nearly 6.3, 6.5, 7.7 and 12 times greater than those of pure flowery WSe_2_, respectively, as shown in Figure 7a. For isoamylol, the response values were as high as 44.5. At a high gas concentration (1000 ppm), the response values for the pure flowery WSe_2_ gas sensor to ethanol, isoamylol, n-butyl alcohol, isopropanol, isobutanol and n-propanol were only 23.2, 41.82, 7.05, 4.43, 25.53 and 8.03, respectively. However, the corresponding response values increased to 178.5, 75, 78, 15, 56.9 and 96.5 for the Au@WSe_2_ NC sensor, respectively, as shown in Figure 7b. The enhancement effect of ethanol, n-butyl alcohol and n-propanol was particularly significant: their response values were nearly 8, 11 and 12 times greater than those of pure flowery WSe_2_, respectively. For ethanol, the response values were as high as 178.5. Therefore, Au@WSe_2_ NCs have a good selectivity for ethanol at a high gas concentration, while they have a good selectivity for isoamylol at a low gas concentration.

**Figure 8:**
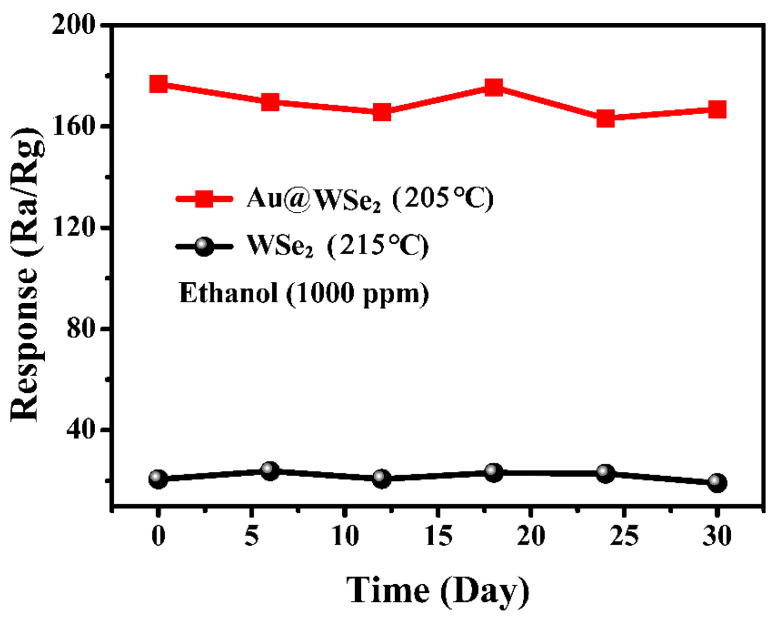
The repeatability and stability of the sensor.

To check the repeatability and stability of the gas sensor, we measured the responses of pure flowery WSe_2_ and Au@WSe_2_ NC sensors to 1000 ppm ethanol, as shown in Figure S3 and Figure 8. It was found that the responses of the two gas sensors were relatively steady, and their repeatability was extremely favorable after a detection period of 30 days.

**Figure 9:**
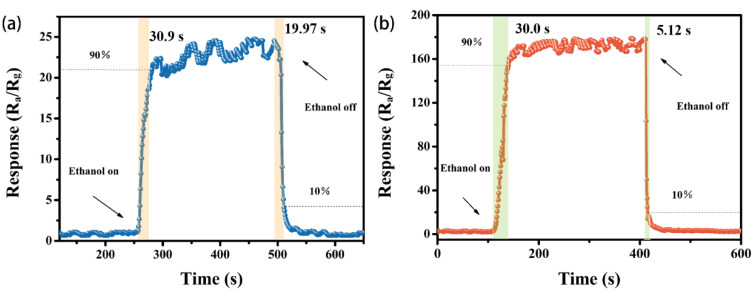
Response and recovery times of pure flowery (**a**) WSe_2_ and (**b**) Au@WSe_2_ NCs for 1000 ppm ethanol at their optimal operating temperatures.

We also investigated the response/recovery characteristics of pure flowery WSe_2_ and Au@WSe_2_ NC sensors for 1000 ppm ethanol at their corresponding optimal operating temperatures, as shown in Figure 9. The response (recovery) time is defined as the time required to rise (fall) to 90% (10%) of the maximum response value. By contrast, the recovery time decreased from 19.97 s to 5.12 s upon exposure to 1000 ppm ethanol with Au NP decoration, which indicates that the Au@WSe_2_ NC sensor has excellent response/recovery behavior.

We also compared Au@WSe_2_ NCs with previously reported sensors, as shown in Table 1. It can be seen that our Au@WSe_2_ NC sensor has a better performance than the reported sensors. Hence, the prepared Au@WSe_2_ NCs have great potential for gas detection (especially for ethanol and isoamylol) in practical applications.

### 3.3. Sensing Mechanism

The sensing mechanism can be described as an adsorption–oxidation–desorption process, which results in a change in the resistance of the sensor. When the gas sensor is exposed to the air, oxygen molecules will be adsorbed on the sensor surface and capture electrons from the conduction band of the semiconductor to form adsorbed oxygen ions ($O_{2\left(ads\right)}^{-}$ or $O_{\left(ads\right)}^{-}$). The reaction process is outlined below:

(1) $$O_{2\left(ads\right)}+e^{-}\;\to {\;O}_{2\left(ads\right)}^{-}\;(3\text{-}1)$$

(2) $$O_{2\left(ads\right)}^{-}+e^{-}\;\to \;2O_{\left(ads\right)}^{-}\;(3\text{-}2)$$

At this time, a depletion layer will form on the surface of the semiconductor, which will significantly decrease the carrier concentration and cause an increase in the resistance.

When the gas sensor is exposed to a reducing gas such as ethanol, the ionized oxygen will react with the reducing gas on the material surface and release electrons back to the conduction band, giving rise to a reduction in the resistance. The reaction can be described as follows.

(3) $$C_{2}H_{5}\mathrm{OH}\;(\mathrm{gas})+O_{\left(ads\right)}^{-}\to \;C_{2}H_{4}O+H_{2}O+e^{-}\;(3\text{-}3)$$

(4) $$C_{2}H_{5}\mathrm{OH}\;(\mathrm{gas})+3O_{2\left(ads\right)}^{-}\to \;2{\mathrm{CO}}_{2}+3H_{2}O+3e^{-}\;(3\text{-}4)$$

**Figure 10:**
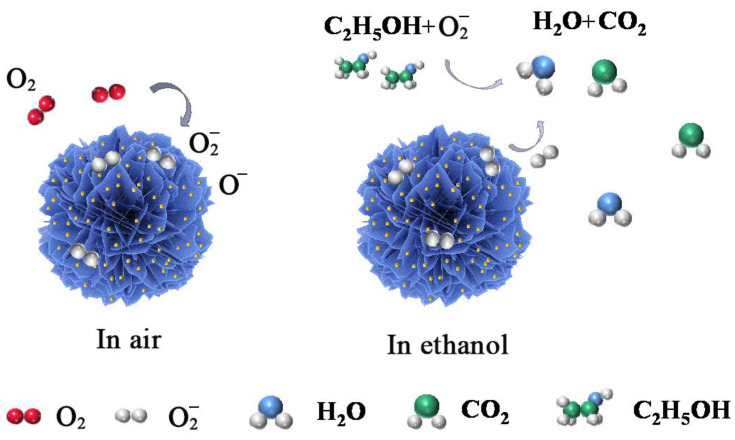
Sensing mechanism of Au@WSe_2_ NCs toward ambient air and ethanol gas.

Compared with pure flowery WSe_2_, the gas sensitivity of Au@WSe_2_ NCs is significantly improved. The mechanism of the enhanced response of Au-NP-decorated flowery WSe_2_ can be explained from the following aspects. (1) The work function of Au (5.1 eV) is larger than that of WSe_2_ (3.61 eV) [35], which results in an electron transfer from WSe_2_ to Au NPs, and a Schottky potential barrier is formed at the interface. The potential barrier results in an increase in the resistance of the gas sensor. Moreover, the accumulation of electrons on the surface of Au NPs leads to the adsorbed oxygen molecules more readily forming oxygen ions ($O_{2\left(ads\right)}^{-}$ or $O_{\left(ads\right)}^{-}$). (2) Because of the chemical spillover effect [36,37], more active adsorption sites are generated on Au NPs, and the formed oxygen ions adsorbed on Au will spread to WSe_2_. When the sensor contacts alcohols, the ionized oxygen will react with the reducing gas on the WSe_2_ surface and release electrons back to WSe_2_, giving rise to a reduction in the resistance and the enhancing the sensor response, as shown in Figure 10. (3) Furthermore, Au NPs promote the reaction on the surface of flowery WSe_2_ and reduce the activation energy for oxygen molecule cleavage, thus reducing the optimal operating temperature [38].

## 4. Conclusions

In this study, we successfully prepared flowery WSe_2_ and Au@WSe_2_ NC sensors and further explored the sensing characteristics of the two devices. The results show that Au-NP-decorated flowery WSe_2_ displays a lower optimal working temperature (205 °C) than that of the pure flowery WSe_2_ sensor (215 °C); meanwhile, the response to alcohols was also significantly enhanced. The flowery WSe_2_ sensor responses to ethanol, n-butyl alcohol and n-propanol at 1000 ppm were 23.20, 7.05 and 8.03, respectively. After being decorated with Au NPs, the responses to ethanol, n-butyl alcohol and n-propanol at 1000 ppm were 178.5, 78.0 and 96.45, respectively, which are 8, 11 and 12 times larger than those of the pure flowery WSe_2_ sensor. At a low gas concentration (100 ppm), the enhancement effect of ethanol, isoamylol, n-butyl alcohol and n-propanol was particularly significant: their response values were nearly 6.3, 6.5, 7.7 and 12 times greater than those of pure flowery WSe_2_, respectively. For isoamylol, the response values were as high as 44.5. Therefore, Au@WSe_2_ NCs have a good selectivity for ethanol at a high gas concentration, while they have a good selectivity for isoamylol at a low gas concentration. The Au/WSe_2_ NC sensor has a short response/recovery time and good repeatability, selectivity and stability for ethanol and isoamylol.

## Figures and Tables

**Table 1:** Comparison of key parameters between reported sensors and Au@WSe_2_ NCs sensor.

Sensing Materials	Target Gas	Operating Temperature (°C)	Concentration (ppm)	Response	Ref.
WSe_2_ nanosheets	Ethanol	RT	30	1.2 ^a^	[23]
WSe_2_ nanosheets WSe_2_ nanosheets	NO_2_ NO_2_	RT RT	10 0.05	5.36 ^a^ 5.06 ^a^	[29] [24]
Flowery CuO	Ethanol	260	1000	4 ^b^	[30]
Flowery TiO_2_	Acetone	330	250	33.72 ^a^	[31]
Flowery WO_3_	NO_2_	90	0.08	152 ^b^	[32]
Flowery SnO_2_	Ethanol	_	100	29.7 ^a^	[33]
WSe_2_@TiO_2_ NCs	Ethanol	RT	100	42.8 ^a^	[23]
Au@CuO NCs	Ethanol	100	1000	95.3 ^b^	[18]
Au@ZnO NCs	Ethanol	125	1000	1.42 ^a^	[34]
Au@SnO_2_ NCs	Ethanol	240	100	23.93 ^a^	[15]
Flowery WSe_2_	Ethanol (isoamylol)	215	1000 (100)	23.2 (6.8)	This work
Au@ flowery WSe_2_	Ethanol (isoamylol)	205	1000 (100)	178.5 (44.5)	This work

^a^ Response = R_a_/R_g_; ^b^ Response = R_g_/R_a_.

## Supplementary Materials

The following supporting information can be downloaded at: https://www.mdpi.com/article/10.3390/nano12234221/s1, Figure S1: (a) The deposited electrodes and (b) covered contact area; Figure S2: The SEM image of an ensemble of the flowery WSe2 nanostructures; Figure S3: The reproducibility test of (a) pure WSe2 and (b) Au@WSe2 based sensors to 1000 ppm ethanol gas at their optimal operating temperature.

## References

[1] Gong H., Hu J., Wang J., Ong C., Zhu F. (2006). Nano-crystalline Cu-doped ZnO thin film gas sensor for CO. Sens. Actuators B Chem..

[2] Tsujita W., Yoshino A., Ishida H., Moriizumi T. (2005). Gas sensor network for air-pollution monitoring. Sens. Actuators B Chem..

[3] Donarelli M., Ottaviano L. (2018). 2D materials for gas sensing applications: A review on graphene oxide, MoS_2_, WS_2_ and phosphorene. Sensors.

[4] Poulopoulos S., Samaras D., Philippopoulos C. (2001). Regulated and unregulated emissions from an internal combustion engine operating on ethanol-containing fuels. Atmos. Environ..

[5] Huang H., Zhou J., Chen S., Zeng L., Huang Y. (2004). A highly sensitive QCM sensor coated with Ag^+^-ZSM-5 film for medical diagnosis. Sens. Actuators B Chem..

[6] Mohammadfam I., Zarei E. (2015). Safety risk modeling and major accidents analysis of hydrogen and natural gas releases: A comprehensive risk analysis framework. Int. J. Hydrogen..

[7] Ko K.Y., Park K., Lee S., Kim Y., Woo W.J., Kim D., Song J.-G., Park J., Kim H. (2018). Recovery improvement for large-area tungsten diselenide gas sensors. ACS Appl. Mater. Inter..

[8] Cheng Y., Wang K., Qi Y., Liu Z. (2022). Chemical vapor deposition method for graphene fiber materials. Acta Phys.-Chim. Sin..

[9] Zheng W., Xiong X.F., Lin R.C., Zhang Z.J., Xu C.H., Huang F. (2018). Balanced Photodetection in One-Step Liquid-Phase-Synthesized CsPbBr_3_ Micro-/Nanoflake Single Crystals. ACS Appl. Mater. Inter..

[10] Sun M., Chou J.-P., Yu J., Tang W. (2017). Effects of structural imperfection on the electronic properties of graphene/WSe_2_ heterostructures. J. Mater. Chem. C.

[11] Jeon D., Kang Y., Kim T. (2020). Observing the layer-number-dependent local dielectric response of WSe_2_ by electrostatic force microscopy. J. Phys. Chem. Lett..

[12] Abbasi A., Sardroodi J.J. (2018). Investigation of the adsorption of ozone molecules on TiO_2_/WSe_2_ nanocomposites by DFT computations: Applications to gas sensor devices. Appl. Surf. Sci..

[13] Zhang S., Li R., Yao Z., Liao P., Li Y., Tian H., Wang J., Liu P., Guo J., Liu K. (2020). Laser annealing towards high-performance monolayer MoS_2_ and WSe_2_ field effect transistors. Nanotechnology.

[14] Sun Y., He N., Wang Y., Yuan Q., Wen D. (2022). Multilevel memory and artificial synaptic plasticity in P(VDF-TrFE)-based ferroelectric field effect transistors. Nano Energy.

[15] Liu Y., Li X., Wang Y., Li X., Cheng P., Zhao Y., Dang F., Zhang Y. (2020). Hydrothermal synthesis of Au@ SnO_2_ hierarchical hollow microspheres for ethanol detection. Sens. Actuators B Chem..

[16] Peng C., Liu Y. (2013). Enhanced acetone sensing characteristics by decorating Au nanoparticles on ZnO flower-like structures. Appl. Phys. A.

[17] Li F., Wang S., Wu Z., Xiong X., Li J., Zhou J., Gao X. (2021). Excellent ethanol sensor based on LaFeO_3_ modified with gold nanoparticles. J. Mater. Sci.-Mater. El..

[18] Cheng M., Li W., Li C., Wang Q., Tan Q., Yang W., Liu Y. (2021). Photochemical sensitive study of Au@CuO flower-like materials. Sens. Actuators B Chem..

[19] Zhang Z.-W., Li Q.-H., Qiao X.-Q., Hou D., Li D.-S. (2019). One-pot hydrothermal synthesis of willow branch-shaped MoS_2_/CdS heterojunctions for photocatalytic H_2_ production under visible light irradiation. Chin. J. Catal..

[20] Rao Z.-M., Xie J.-Y., Liu L.-J., Zeng Y.-Y. (2007). Cai, W.-L. Study on gaseous methanol sensor utilizing cataluminescence of TiO_2_-Y_2_O_3_ powder. Acta Chim. Sin..

[21] Mi H., Zhou Q., Zeng W. (2021). A density functional theory study of the adsorption of Cl_2_, NH_3_, and NO_2_ on Ag_3_-doped WSe_2_ monolayers. Appl. Surf. Sci..

[22] Yu B., Zheng B., Wang X., Qi F., He J., Zhang W., Chen Y. (2017). Enhanced photocatalytic properties of graphene modified few-layered WSe_2_ nanosheets. Appl. Surf. Sci..

[23] Pan W., Zhang Y., Zhang D. (2020). Self-assembly fabrication of titanium dioxide nanospheres-decorated tungsten diselenide hexagonal nanosheets for ethanol gas sensing application. Appl. Surf. Sci..

[24] Guo R., Han Y., Su C., Chen X., Zeng M., Hu N., Su Y., Zhou Z., Wei H., Yang Z. (2019). Ultrasensitive room temperature NO_2_ sensors based on liquid phase exfoliated WSe_2_ nanosheets. Sens. Actuators B Chem..

[25] Selin C. (2007). Expectations and the Emergence of Nanotechnology. Sci. Technol. Hum. Val..

[26] Xu J., Wei Z., Zhang S., Wang X., Wang Y., He M., Huang K. (2021). Hierarchical WSe_2_ nanoflower as a cathode material for rechargeable Mg-ion batteries. J. Colloid Interface Sci..

[27] Chen W., Liu Y., Qin Z., Wu Y., Li S., Gong N. (2016). Improved ethanediol sensing with single Yb ions doped SnO_2_ nanobelt. Ceram. Int..

[28] Yan X., Yang W., Li C., Liu L., Liu Y. (2021). CdS Micrometer Hollow Spheres for Detecting Alcohols Except Methanol with Strong Anti-interference Ability. ACS Omegar..

[29] Yang C., Xie J., Lou C., Zheng W., Liu X., Zhang J. (2021). Flexible NO_2_ sensors based on WSe_2_ nanosheets with bifunctional selectivity and superior sensitivity under UV activation. Sens. Actuators B Chem..

[30] Yang C., Xiao F., Wang J., Su X. (2015). 3D flower-and 2D sheet-like CuO nanostructures: Microwave-assisted synthesis and application in gas sensors. Sens. Actuators B Chem..

[31] Ge W., Jiao S., Chang Z., He X., Li Y. (2020). Ultrafast response and high selectivity toward acetone vapor using hierarchical structured TiO_2_ nanosheets. ACS Appl. Mater. Inter..

[32] Wang C., Sun R., Li X., Sun Y., Sun P., Liu F., Lu G. (2014). Hierarchical flower-like WO_3_ nanostructures and their gas sensing properties. Sens. Actuators B Chem..

[33] Liu Y., Jiao Y., Zhang Z., Qu F., Umar A., Wu X. (2014). Hierarchical SnO_2_ nanostructures made of intermingled ultrathin nanosheets for environmental remediation, smart gas sensor, and supercapacitor applications. ACS Appl. Mater. Inter..

[34] Wongrat E., Chanlek N., Chueaiarrom C., Samransuksamer B., Hongsith N., Choopun S. (2016). Low temperature ethanol response enhancement of ZnO nanostructures sensor decorated with gold nanoparticles exposed to UV illumination. Sens. Actuators A-Phys..

[35] Chen C.-H., Wu C.-L., Pu J., Chiu M.-H., Kumar P., Takenobu T., Li L.-J. (2014). Hole mobility enhancement and p-doping in monolayer WSe_2_ by gold decoration. 2D Mater..

[36] Lee J.-S., Katoch A., Kim J.-H., Kim S.S. (2016). Effect of Au nanoparticle size on the gas-sensing performance of *p*-CuO nanowires. Sens. Actuators B Chem..

[37] Mabelet L., Malonda-Boungou B., Mabiala-Poaty H., Raji A., M’Passi-Mabiala B. (2020). Energetics, electronic and magnetic properties of monolayer WSe_2_ doped with pnictogens, halogens and transition-metal (4d, 5d) atoms: An ab-initio study. Physica E.

[38] Zeb S., Sun G., Nie Y., Cui Y., Jiang X. (2020). Synthesis of highly oriented WO_3_ nanowire bundles decorated with Au for gas sensing application. Sens. Actuators B Chem..

