# Supplementary material for: Enhanced Gas Sensitivity of Au-Decorated Flowery WSe2 Nanostructures
Source: Nanomaterials (Basel). 2022 Nov 27;12(23):4221. doi: 10.3390/nano12234221 (PMC9738013; doi:10.3390/nano12234221)
Supplement: Supplementary file 1 [file nanomaterials-12-04221-s001.zip › nanomaterials-2044961-supplementary.pdf]

nanomaterials

Supplementary Materials

Enhanced Gas Sensitivity of Au-Decorated Flowery WSe_2 Nanostructures

Xia Zhang [1], Qiuhong Tan [1,2,3,*], Qianjin Wang [1,2,3,*], Peizhi Yang [3] and Yingkai Liu [1,2,3]

1 College of Physics and Electronic Information, Yunnan Normal University, Yunnan Kunming 650500, China
2 Yunnan Provincial Key Laboratory for Photoelectric Information Technology, Yunnan Normal University, Yunnan Kunming 650500, China
3 Key Laboratory of Advanced Technique & Preparation for Renewable Energy Materials, Ministry of Education, Yunnan Normal University, Kunming 650500, China
* Correspondence: tanqiuhong1@126.com (Q.T.); qjwang@xtu.edu.cn (Q.W.)

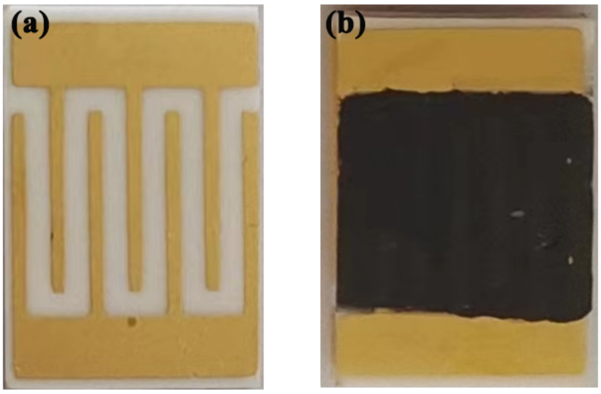

Figure S1. (**a**) The deposited electrodes and (**b**) covered contact area.

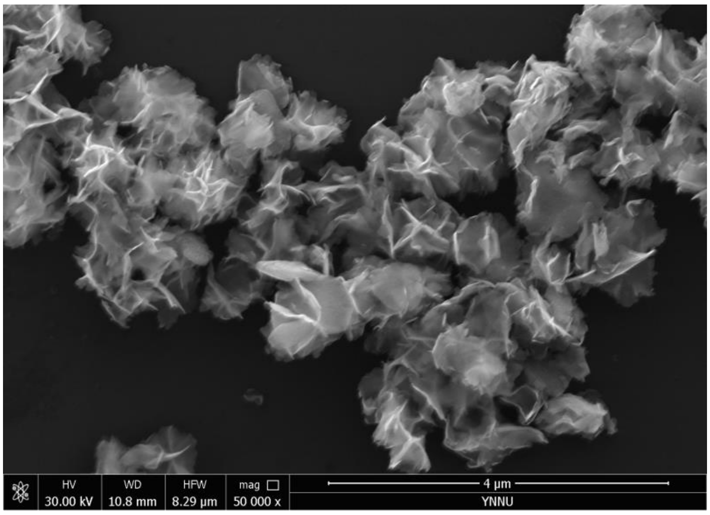

Figure S2. The SEM image of an ensemble of the flowery WSe_2 nanostructures.

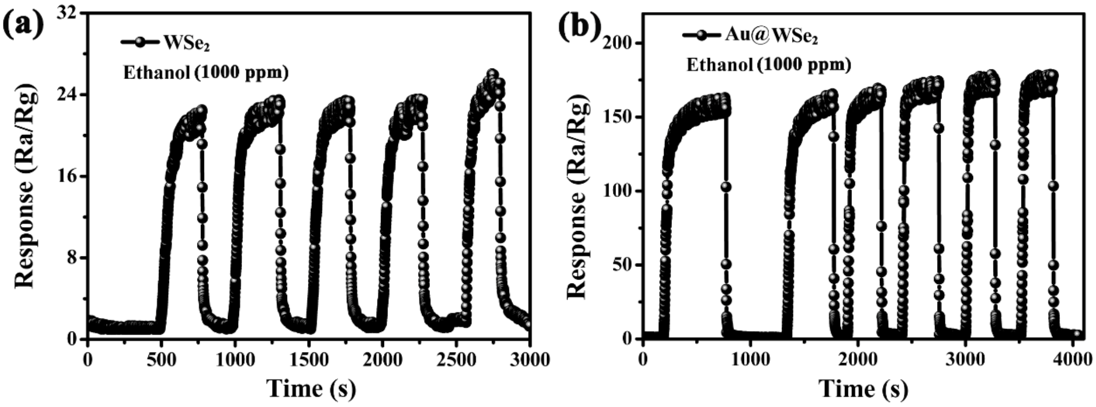

Figure S3. The reproducibility test of (**a**) pure WSe_2 and (**b**) Au@WSe_2 based sensors to1000 ppm ethanol gas at their optimal operating temperature.
